# Supplementary material for: Magnetic Iron Oxide Nanoparticles Coated by Coumarin-Bound Copolymer for Enhanced Magneto- and Photothermal Heating and Luminescent Thermometry
Source: Nanomaterials (Basel). 2024 May 22;14(11):906. doi: 10.3390/nano14110906 (PMC11173931; doi:10.3390/nano14110906)
Supplement: Supplementary file 1 [file nanomaterials-14-00906-s001.zip › nanomaterials-3011778-supplementary.pdf]

Electronic Supplementary Materials

Magnetic IONP Nanoparticles Coated by Coumarin-bound Copolymer for Enhanced Magneto- and Photothermal Heating and Luminescent Thermometry

Alexiane Féron, Sylvain Catrouillet,* Saad Sene, Gautier Félix, Belckacem Tarek Benkhaled, Vincent Lapinte, Yannick Guari* and Joulia Larionova*

ICGM, Univ. Montpellier, CNRS, ENSCM, Montpellier, France.

E-mail : alexiane.feron@etu.umontpellier.fr (A.F.); sylvain.catrouillet@umontpellier.fr (S.C.); saad.sene@umontpellier.fr (S.S.); gautier.felix@umontpellier.fr (G.F.); belkacem-tarek.benkhaled@umontpellier.fr (B.T.B.); Vincent.lapinte@umontpellier.fr (V.L.); yannick.guari@umontpellier.fr (Y.G.); joulia.larionova@umontpellier.fr (J.L.)

Contents

Figure S1. ^{1}H NMR spectra of Coum-C_{11}-$PPhOx_{27}$-$PMOx_{59}$, Coum-C_{11}-$PButOx_8$-$PMOx_{42}$ and $PPhOx_{27}$-$PMOx_{57}$.......................... 3

Figure S2. GPC trace of a) $PPhOx_{27}$-$PMOx_{57}$, b) Coum-C_{11}-$PPhOx_{27}$-$PMOx_{59}$, and c) Coum-C_{11}-$PButOx_8$-$PMOx_{42}$.......................... 3

Figure S3. Schematic representation and representative photographs for different steps in the preparation of Coum-C_{11}-$PPhOx_{27}$-$PMOx_{59}$ (P1) amphiphilic copolymer micelles. 3

Figure S4. Schematic representation of Coum-C_{11}-$PPhOx_{27}$-$PMOx_{59}$ amphiphilic copolymer dimerization (P2) under UV irradiation.4

Figure S5. a) Correlation curve and b) Dynamic light scattering (DLS) for Coum-C_{11}-$PPhOx_{27}$-$PMOx_{59}$ amphiphilic copolymer before P1 (orange line) and after dimerization P2 under UV irradiation (blue line), c) TEM image for Coum-C_{11}-$PPhOx_{27}$-$PMOx_{59}$ copolymer after dimerization (P2) showing the micelles formation. 5

Figure S6. Dynamic light scattering (DLS) for copolymers P3 (a) and P4 (b); c) Representative TEM image for P4 showing cylindrical/vermicular shape of micelles. 6

Figure S7. a) Emission spectra recorded in the temperature range 20 and 60 °C in water for amphiphilic copolymers: a) P3 (λ_{ex} = 323 nm), b) P4 (λ_{ex} = 351 nm), c) P5 (λ_{ex} = 336 nm). 7

Figure S8. Temperature dependence of the luminescent intensity I_{384} with the integrated area 350 – 500 nm in the temperature range 20 – 60 °C for: a) P3, b) P4, c) P5. The solid line represents a linear fitting. Inset: temperature dependence of S_r. 8

Figure S9. Dynamic light scattering (DLS) measurements for IONP@Coum-C_{11}-$PPhOx_{27}$-$PMOx_{59}$ nanoparticles after the first (black curve) and the third (red curve) washing cycles. 9

Figure S10. Size distribution for IONP/OA/OAm (*n = 300*). 9

Figure S11. Simulated light density power of the 808 nm LASER used for the fit of the photothermal experiment for the 0.637 mg/mL (a) and 0.478 mg/mL (b) concentrations. 10

Figure S12. Simulated temperature obtained during the fit of the photothermal experiment for the 0.637 mg/mL concentration at 10 s (a) and 900 s (c) and the 0.478 mg/mL concentration at 10 s (b) and 900 s (d).11

Figure S13. Simulated natural convection obtained during the fit of the photothermal experiment for the 0.637 mg/mL concentration at 10 s (a) and 900 s (c) and the 0.478 mg/mL concentration at 10 s (b) and 900 s (d).12

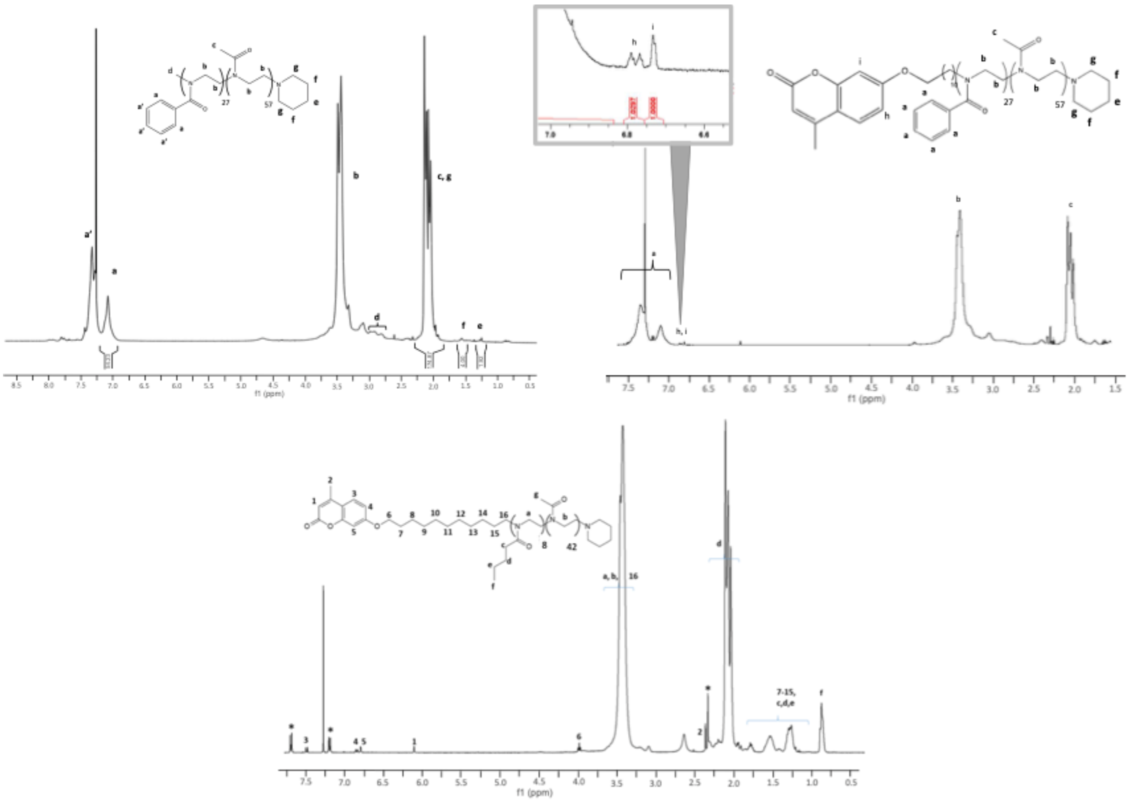

Figure S1. ^{1}H NMR spectra of Coum-C_{11}-PPhOx$_{27}$-PMOx$_{59}$, Coum-C_{11}-PButOx$_{8}$-PMOx$_{42}$ and PPhOx$_{27}$-PMOx$_{57}$.

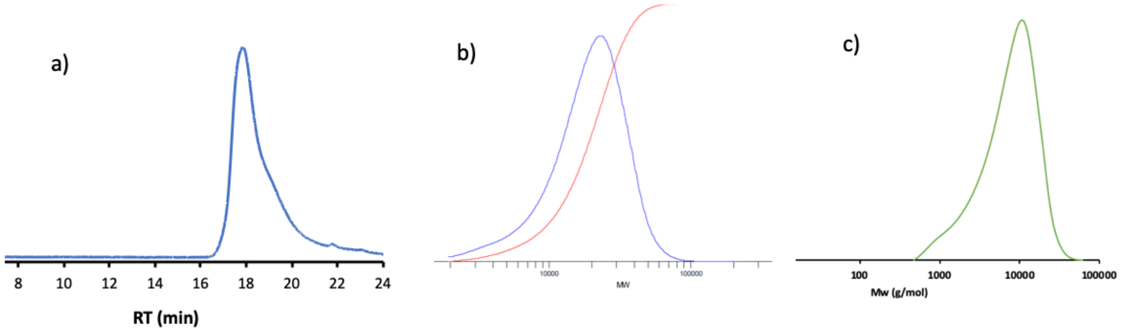

Figure S2. GPC trace of a) PPhOx$_{27}$-PMOx$_{57}$, b) Coum-C_{11}-PPhOx$_{27}$-PMOx$_{59}$, and c) Coum-C_{11}-PButOx$_{8}$-PMOx$_{42}$.

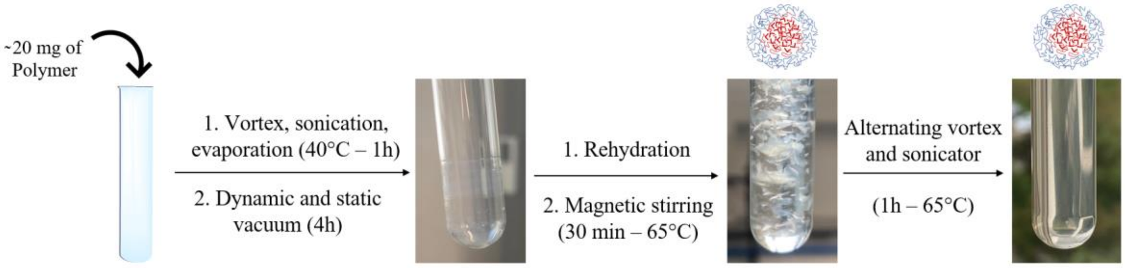

Figure S3. Schematic representation and representative photographs for different steps in the preparation of Coum-C_{11}-PPhOx$_{27}$-PMOx$_{59}$ (P1) amphiphilic copolymer micelles.

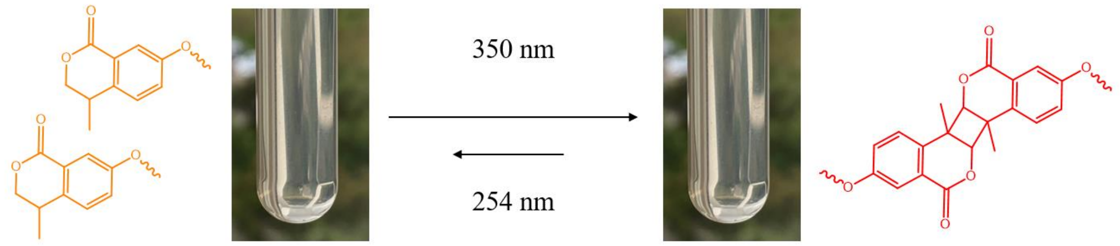

Figure S4. Schematic representation of Coum-C_{11}-$PPhOx_{27}$-$PMOx_{59}$ amphiphilic copolymer dimerization (P2) under UV irradiation.

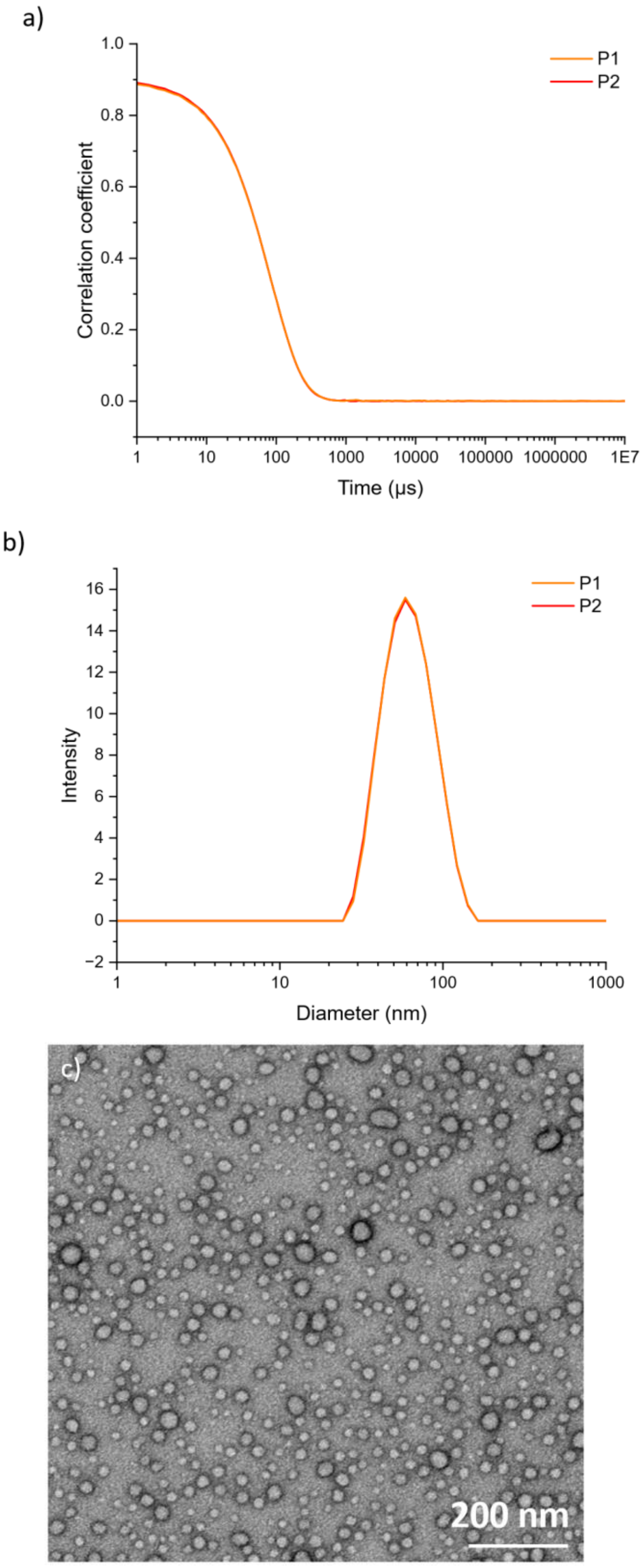

Figure S5. a) Correlation curve and b) Dynamic light scattering (DLS) for Coum-C_{11}-$PPhOx_{27}$-$PMOx_{59}$ amphiphilic copolymer before P1 (orange line) and after dimerization P2 under UV irradiation (blue line), c) TEM image for Coum-C_{11}-$PPhOx_{27}$-$PMOx_{59}$ copolymer after dimerization (P2) showing the micelles formation.

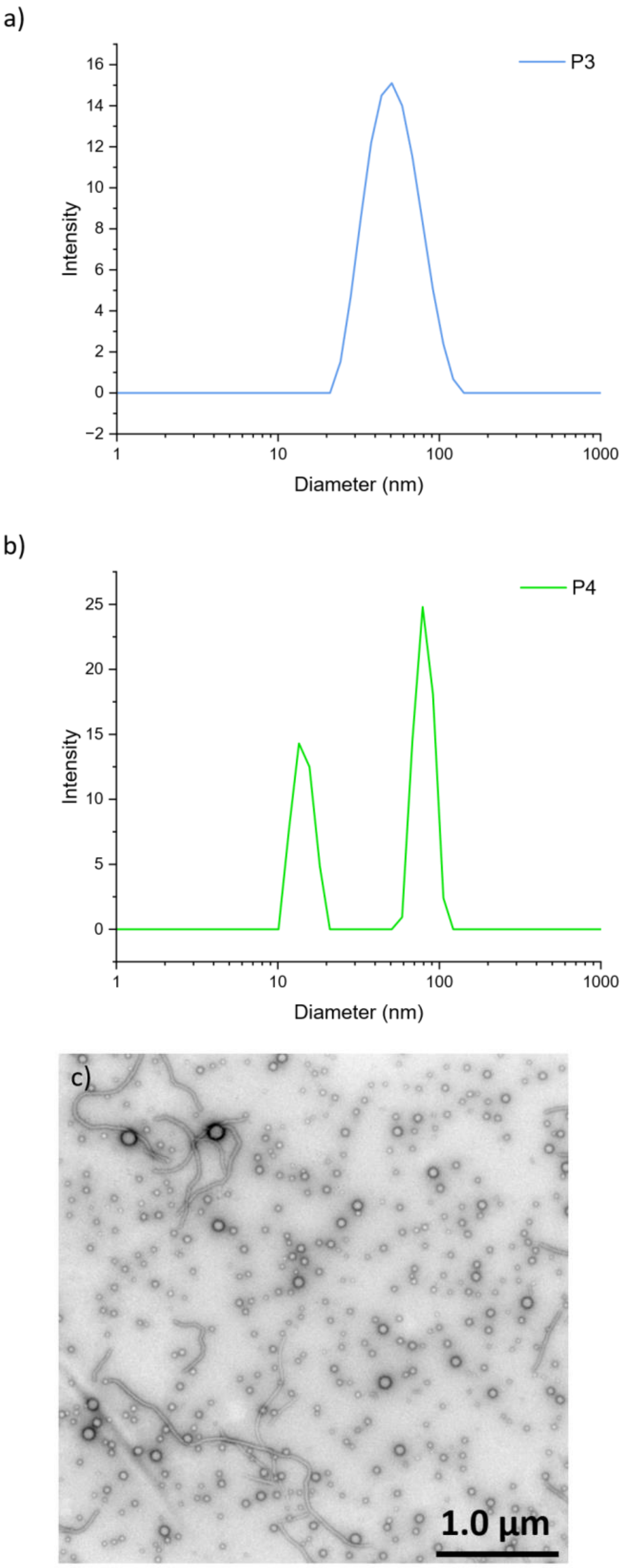

Figure S6. Dynamic light scattering (DLS) for copolymers P3 (a) and P4 (b); c) Representative TEM image for P4 showing cylindrical/vermicular shape of micelles.

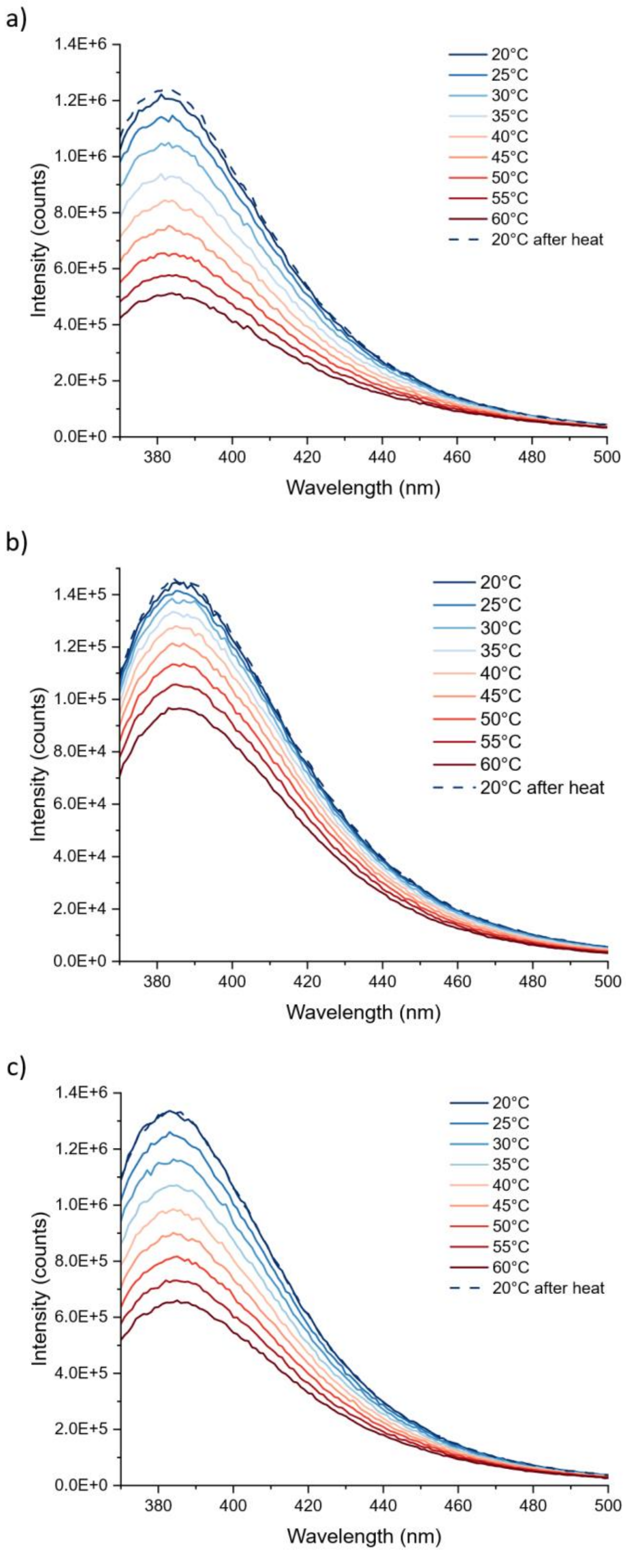

Figure S7. a) Emission spectra recorded in the temperature range 20 and 60 °C in water for amphiphilic copolymers: a) P3 (λ_{ex} = 323 nm), b) P4 (λ_{ex} = 351 nm), c) P5 (λ_{ex} = 336 nm).

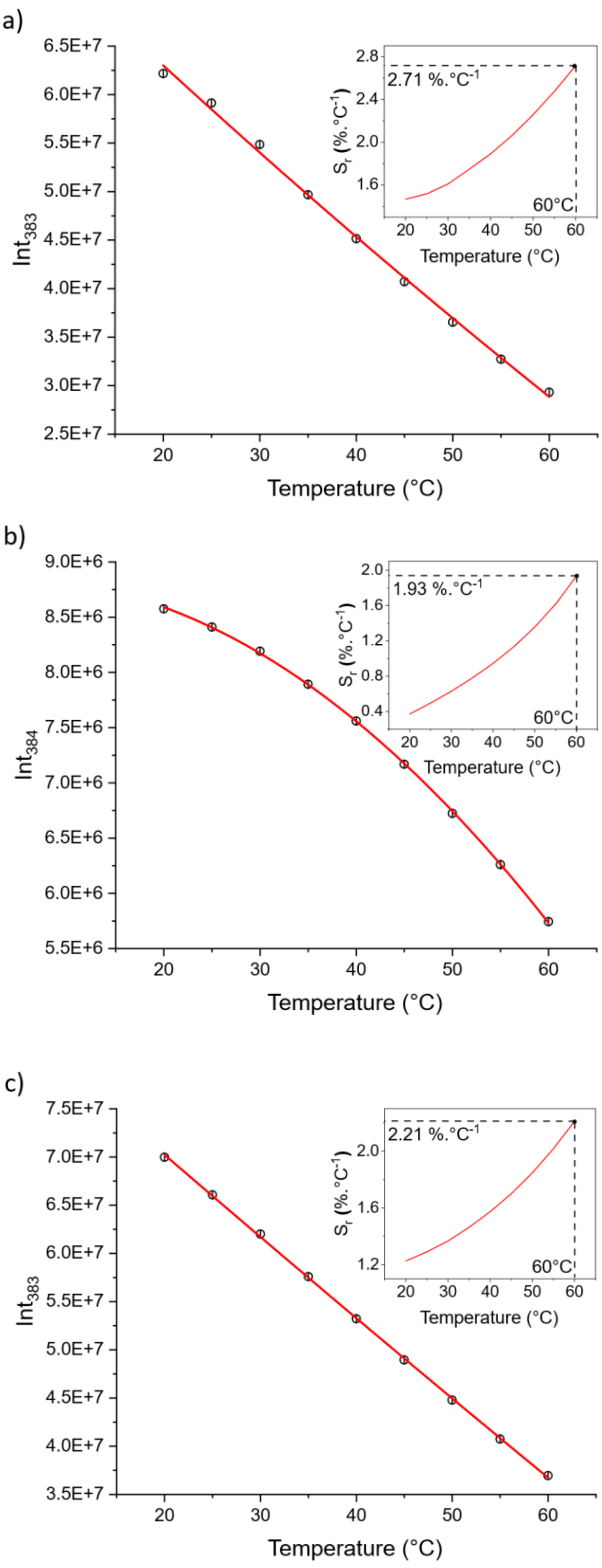

Figure S8. Temperature dependence of the luminescent intensity I_{384} with the integrated area 350 – 500 nm in the temperature range 20 – 60 °C for: a) P3, b) P4, c) P5. The solid line represents a linear fitting. Inset: temperature dependence of S_r.

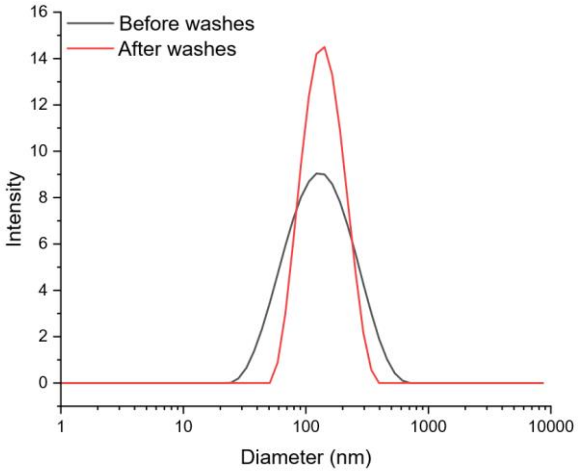

Figure S9. Dynamic light scattering (DLS) measurements for IONP@Coum-C_{11}-$PPhOx_{27}$-$PMOx_{59}$ nanoparticles after the first (black curve) and the third (red curve) washing cycles.

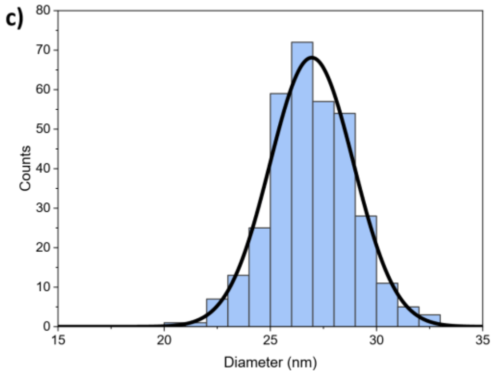

Figure S10. Size distribution for IONP/OA/OAm (*n = 300*).

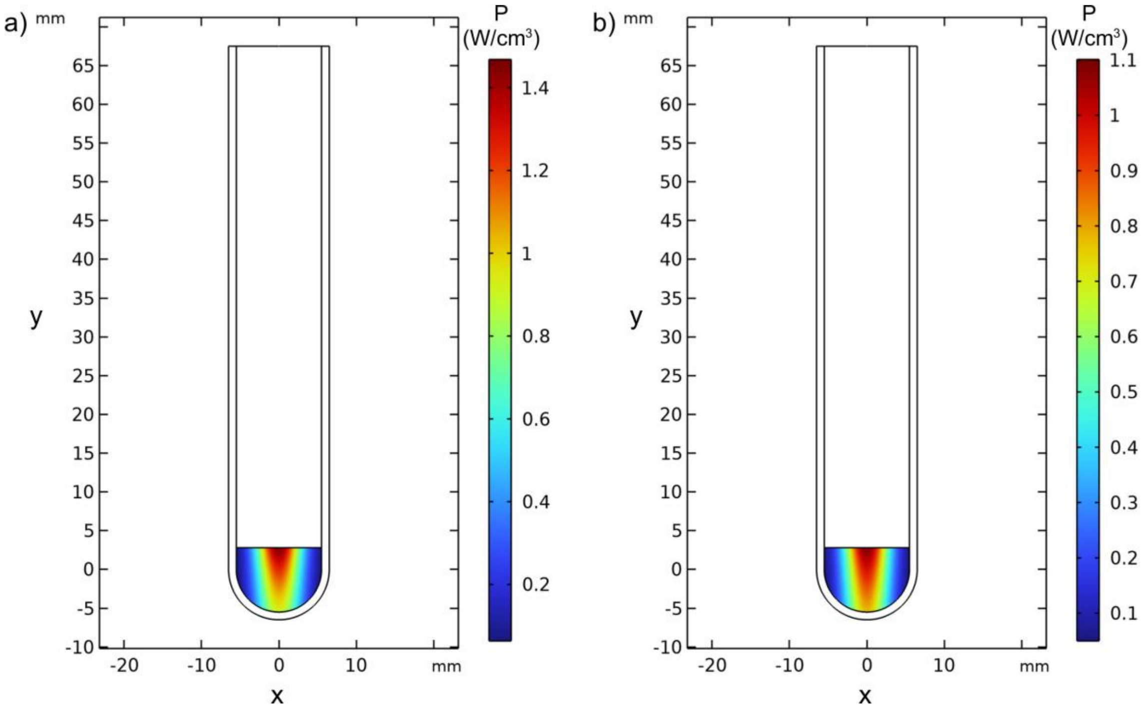

Figure S11. Simulated light density power of the 808 nm LASER used for the fit of the photothermal experiment for the 0.637 mg/mL (a) and 0.478 mg/mL (b) concentrations.

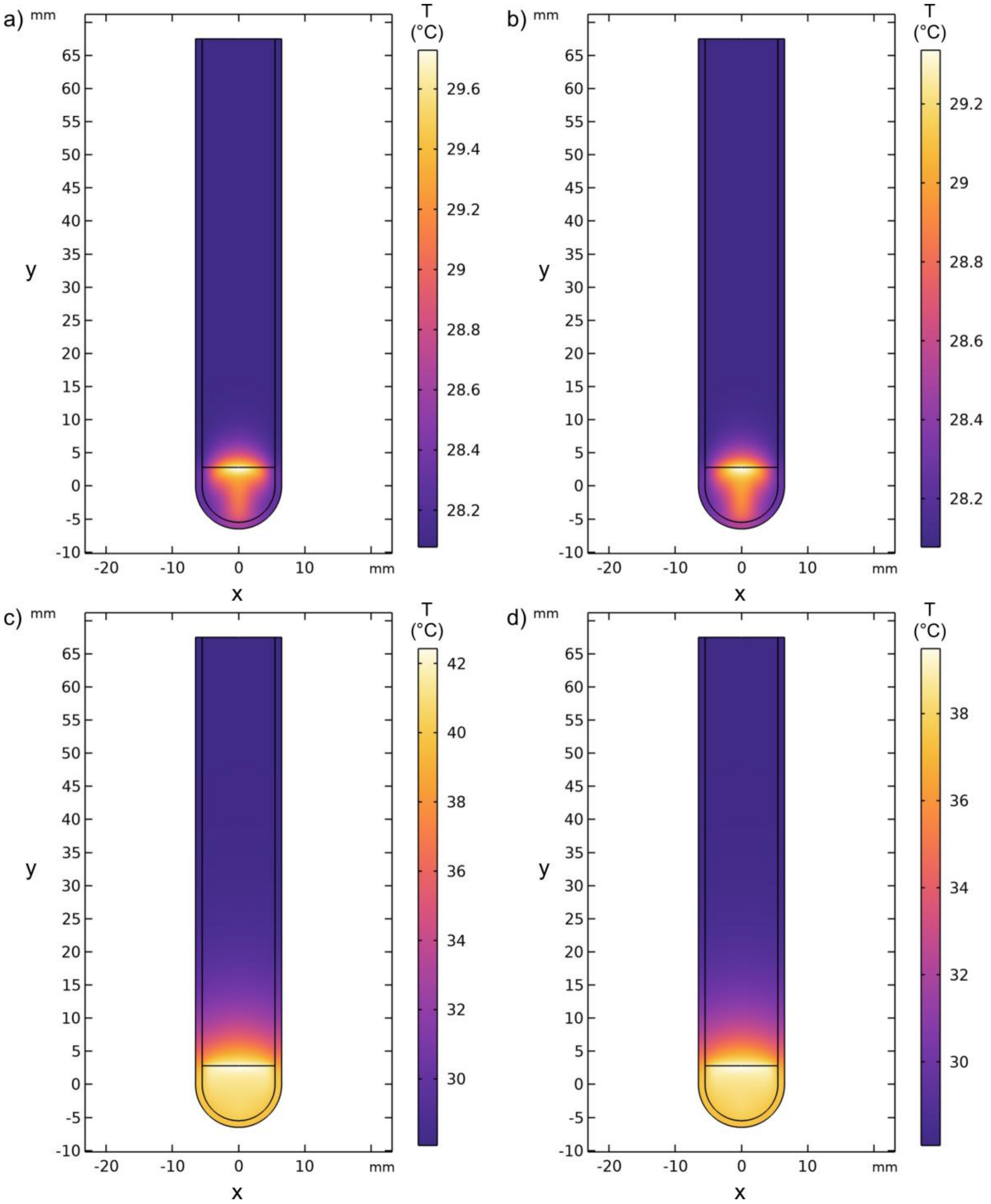

Figure S12. Simulated temperature obtained during the fit of the photothermal experiment for the 0.637 mg/mL concentration at 10 s (a) and 900 s (c) and the 0.478 mg/mL concentration at 10 s (b) and 900 s (d).

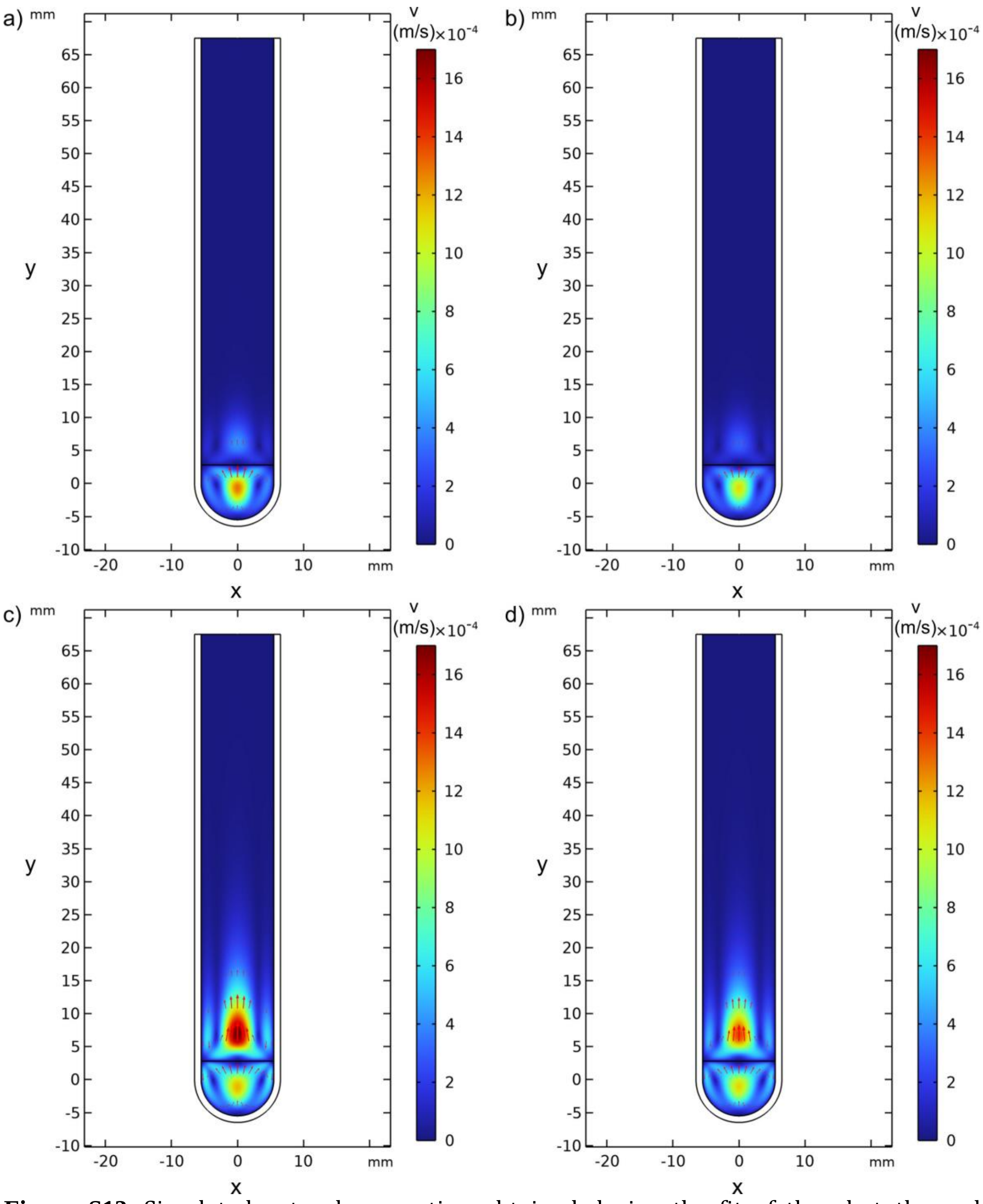

Figure S13. Simulated natural convection obtained during the fit of the photothermal experiment for the 0.637 mg/mL concentration at 10 s (a) and 900 s (c) and the 0.478 mg/mL concentration at 10 s (b) and 900 s (d).
